# Genetic alterations analysis in prognostic stratified groups identified TP53 and ARID1A as poor clinical performance markers in intrahepatic cholangiocarcinoma

**DOI:** 10.1038/s41598-018-25669-1

**Published:** 2018-05-08

**Authors:** Michele Simbolo, Caterina Vicentini, Andrea Ruzzenente, Matteo Brunelli, Simone Conci, Matteo Fassan, Andrea Mafficini, Borislav Rusev, Vincenzo Corbo, Paola Capelli, Emilio Bria, Serena Pedron, Giona Turri, Rita T. Lawlor, Giampaolo Tortora, Claudio Bassi, Alfredo Guglielmi, Aldo Scarpa

**Affiliations:** 1 ARC-Net Research Centre, University and Hospital Trust of Verona, Verona, Italy; 2 Department of Diagnostics and Public Health, Section of Anatomical Pathology, University and Hospital Trust of Verona, Verona, Italy; 3 Department of Surgery, General and Hepatobiliary Surgery, University Hospital G.B. Rossi, University of Verona, Verona, Italy; 4 Department of Medicine, U.O.C Oncology, University and Hospital Trust of Verona, Verona, Italy; 5 Department of Surgery and Oncology, Pancreas Institute, University of Verona, Verona, Italy; 6 Present Address: Department of Medicine (DIMED), Surgical Pathology and Cytopathology Unit, University of Padua, Padua, Italy; 7 Medical Oncology, Università Cattolica del Sacro Cuore, Fondazione Policlinico ‘A. Gemelli’, Roma, Italy

## Abstract

The incidence and mortality rates of intrahepatic cholangiocarcinoma have been rising worldwide. Few patients present an early-stage disease that is amenable to curative surgery and after resection, high recurrence rates persist. To identify new independent marker related to aggressive behaviour, two prognostic groups of patient were selected and divided according to prognostic performance. All patients alive at 36 months were included in good prognostic performers, while all patients died due to disease within 36 months in poor prognostic performers. Using high-coverage target sequencing we analysed principal genetic alterations in two groups and compared results to clinical data. In the 33 cases included in poor prognosis group, *TP53* was most mutated gene (*p* = 0.011) and exclusively present in these cases. Similarly, *ARID1A* was exclusive of this group (*p* = 0.024). *TP53* and *ARID1A* are mutually exclusive in this study. Statistical analysis showed mutations in *TP53* and *ARID1A* genes and amplification of *MET* gene as independent predictors of poor prognosis (*TP53*, *p* = 0.0031, *ARID1A*, *p* = 0.0007, *MET*, *p* = 0.0003 in Cox analysis). LOH in *PTEN* was also identified as marker of disease recurrence (*p* = 0.04) in univariate analysis. This work improves our understanding of aggressiveness related to this tumour type and has identified novel prognostic markers of clinical outcome.

## Introduction

Intrahepatic cholangiocarcinoma (ICC) is the second most common primary hepatic malignancy after hepatocellular carcinoma, and accounts for 10–20% of primary liver cancers^1–3^. The incidence and mortality rates of ICC have been rising worldwide in the past decade^2,3^. Moreover, only 10–20% of patients present with early-stage disease that is amenable to curative surgery^4,5^ and after resection, a high recurrence rate of 50–60% makes for a dismal five-year overall survival (OS) of only 30%^5–8^. As for patients with locally advanced or metastatic disease that constitute the bulk of newly diagnosed cases, even with the standard treatment of gemcitabine and cisplatin combination chemotherapy their median survival remains less than one year^9^. Molecular analyses suggested that the observed heterogeneity in prognosis and response to treatments could be attributed to the underlying differential alteration of the molecular mechanisms that drive crucial differences in cancer aggressiveness and treatment outcomes^10,11^.

In this study, we composed two groups of intrahepatic cholangiocarcinomas patients with different prognostic performance. Using high-coverage targeted sequencing (HCTS), we investigated somatic mutations and copy number status of a large number of genes that have been identified as frequently altered by previous studies^12–15^ to uncover the molecular features characterizing the more aggressive subpopulation of this tumour type.

## Results

### Patient characteristics

A retrospective series (1990–2013) of 66 surgically-resected primary intrahepatic cholangiocarcinomas (ICC) was retrieved from the ARC-Net Biobank at Verona University Hospital. Clinico-pathological characteristics of the sample cohort along with the molecular analyses conducted in this study are reported in Table 1. We selected patients according to the following criteria: i) minimum follow-up of 3 years; ii) availability of material for research; iii) no preoperative therapy received. All patient enrolled were subjected to surgical resection of primary tumour and presented negative liver fluke status. Hepatitis B or C virus (HBV/HCV) infection was present in 4/66 (6.1%) cases while liver fluke status was negative for all patients. Liver cirrhosis was present in 7/66 (10.6%) patients. Overall survival (OS) was available for all patients, while disease free survival (DFS) for 42 patients. The whole cohort of patients showed a median follow up of 40.7 months (range 2.0–152.7).

**Table 1:** Clinico-pathological features of 66 intrahepatic cholangiocarcinomas included in the study.

Clinico-pathological features	Poor 33	Good 33	Total 66	p-value*
Sex	9F 24M	16F 17M	25F 41M	0.13
Age	67.8 ± 9.7	60.6 ± 13.6	65.0 ± 11.8	0.334^
Dimension (cm)	6.7 ± 4.4	6.1 ± 2.6	6.4 ± 3.6	0.478^
Median Follow-up (months)	17.1	61.3	40.7	**<0**.**0001**^**#**^
Range	(2.0–40.0)	(40.7–152.7)	(2.0–152.7)	
Median DFS (months)	7.4	61.7	14.2	**<0**.**0001**^**#**^
Range	(2.8–26.4)	(34.0–152.7)	(6.3–152.7)	
NA	10	14	24	
**Recurrence**				
1	22	11	33	**0**.**006**
0	1	8	9	
NA	10	14	24	
**TNM Stage**				
1	2	10	12	0.084
2	12	8	20	
3	7	5	12	
4	12	10	22	
**Grade**				
1	3	4	7	0.226
2	19	24	43	
3	11	5	16	
**R**				
2	1	0	1	0.278
1	5	2	7	
0	27	31	58	
**Liver fluke status**				
Positive	0	0	0	—
Negative	33	33	66	
**HBV/HCV infection**				
Positive	4	0	4	0.11
Negative	29	33	62	
**Primary sclerosing cholangitis**				
Positive	0	0	0	—
Negative	33	33	66	
**Biliary stone disease**				
Positive	0	1	1	—
Negative	33	32	65	
**Liver cirrhosis**				
Positive	7	0	7	0.011
Negative	26	33	59	
**Multiple nodes**				
Present	16	10	26	0.207
Absent	17	23	40	
**Presence of BIN**				
Present	8	3	11	0.185
Absent	25	30	55	
**Vascular invasion**				
Present	28	20	48	0.051
Absent	5	13	18	
**Perineural invasion**				
Present	17	13	30	0.458
Absent	16	20	36	

Note: BIN, biliary intraepithelial neoplasia; DFS, disease free survival; NA, not available; R, resection margins. *Fisher exact test for multiple comparison was used as appropriated; ^Unpaired t-test; # Kaplan-Maier analysis.

### Patient grouping according to prognostic performance

**Figure 1:**
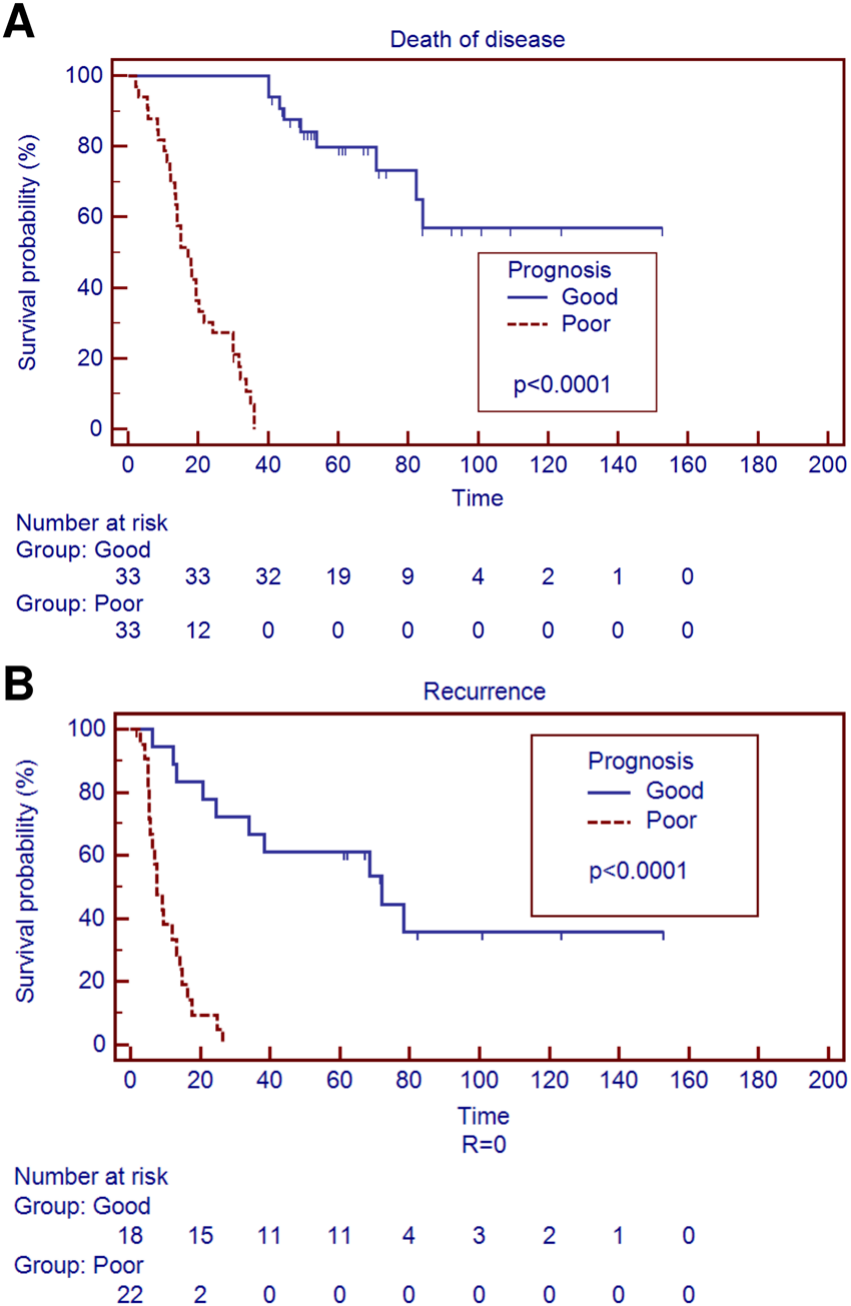
Grouping according to prognosis of 66 intrahepatic cholangiocarcinomas. (**A**) Overall survival significantly divided 66 intrahepatic cholangiocarcinoma in two prognostic performers group according to clinical outcome (*p* < 0.0001). (**B**) Prognostic grouping also divided intrahepatic cholangiocarcinomas characterized by negative resection margins in two different groups (*p* < 0.0001).

For the present study, the sixty-six patients were divided into two groups based on clinical performance: patients alive at least 36 months (33 patients) were defined as good prognostic performers (GP group) and patients dead of disease within 36 months (33 patients) as poor prognostic performers (PP group). The PP group had a median follow up of 17.1 months (range 2.0–36.0) whereas the GP group had a median follow up of 61.3 (range 40.7–152.7). Clinicopathological characteristics of these two groups and of the whole cohort are shown in Table 1 and further detailed in Supplementary Table S1. Patient’s stratification is illustrated in Fig. 1. A higher rate of disease recurrence (*p* = 0.0006; Table 1) and a shorter time to recurrence among patients with negative resection margins (*p* < 0.0001; Fig. 1B) was observed in the PP group. Moreover, presence of liver cirrhosis was observed only in 7 patients of the PP group (*p* = 0.011). No differences were found for the other variables.

### Molecular features according to prognosis

DNA of tumour/normal matched samples from all cases was successfully amplified in multiplex PCR for 90 relevant genes chosen on the basis of published ICC sequencing studies, and an adequate library for HCTS was obtained. The mean read length was 109 bases and a mean coverage depth of 2537x was achieved, with 87.9% target bases covered more than 100x. A minimum coverage of 20x was obtained in all cases. More specifically, HCTS yielded an average coverage of 1347x (40–3874x) in normal samples and 3728x (259–24725X) in tumour samples. Further detail of coverage depth per sample is reported in Supplementary Table S2.

**Figure 2:**
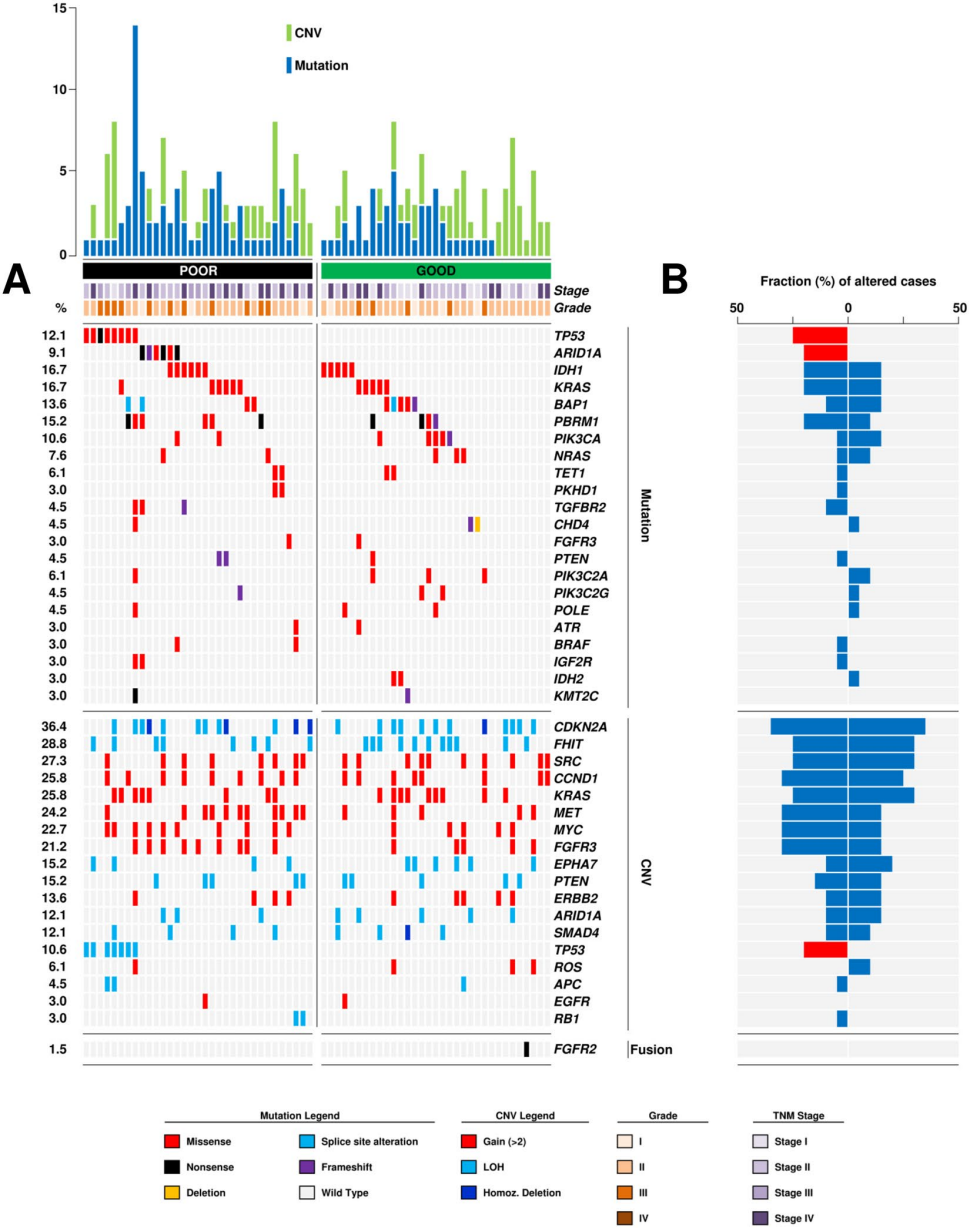
Genetic alterations distinguished two prognostic performer groups. (**A**) Cases are grouped according to prognostic group (poor and good) previous defined by clinical outcome. The upper histogram shows the number of mutations (blue) and CNV (green) in recurrently altered genes for each sample. The central matrix shows 22 genes that were mutated in at least two cases of the whole cohort, 18 genes altered in copy number and 3 genes shown rearrangement; alterations are annotated by different colour according to their impact on the gene product as illustrated in panel below. The number on the left reports the alteration frequency of each gene expressed as a percentage. (**B**) Fraction (%) of cases altered for each gene is represented by blue box or red box (when a statistical significant distribution was observed) according to prognostic group (poor prognosis on the left; good prognosis on the right).

The most frequently altered genes are shown in Fig. 2, distribution and frequencies of mutations in all 90 genes analysed are detailed in Table 2 and Supplementary Table S3. Copy number variations **(**CNVs) were assessed in 18 genes and the results are reported in Table 3. FISH validation of representative cases affected by loss of heterozygosity (LOH) and rearrangement of *FGFR2* are illustrated in Supplementary Figures S1 and S2 respectively.

**Table 2:** Detailed mutational prevalence analysis of 66 intrahepatic cholangiocarcinomas. Related to Fig. 2.

Gene	Poor		Good		Total		Mutation type					p-value*
	33	[%]	33	[%]	66	[%]	M	N	S	D	F	
*ALK*	1	[3.0]			1	[1.5]	1					—
*APC*			1	[3.0]	1	[1.5]	1					—
*ARAF*			1	[3.0]	1	[1.5]	1					—
*ARID1A*	6	[18.2]			6	[9.1]	2	3			1	**0.024**
*ARID1B*	1	[3.0]			1	[1.5]	1					—
*ATR*	1	[3.0]	1	[3.0]	2	[3.0]	2					—
*BAP1*	4	[12.1]	5	[15.2]	9	[13.6]	5		3		1	—
*BRAF*	2	[6.0]			2	[3.0]	2					—
*CHD4*	1	[3.0]	2	[6.0]	3	[4.5]	1			1	1	—
*ERBB3*	1	[3.0]			1	[1.5]	1					—
*ERBB4*	1	[3.0]			1	[1.5]	1					—
*FBXW7*	1	[3.0]			1	[1.5]	1					—
*FGFR3*	1	[3.0]	1	[3.0]	2	[3.0]	2					—
*HRAS*	1	[3.0]			1	[1.5]	1					—
*IDH1*	6	[18.2]	5	[15.2]	11	[16.7]	11					—
*IDH2*			2	[6.0]	2	[3.0]	2					—
*IGF2R*	2	[6.0]			2	[3.0]	2					—
*KDR*	1	[3.0]			1	[1.5]	1					—
*KIT*	1	[3.0]			1	[1.5]	1					—
*KMT2C*	1	[3.0]	1	[3.0]	2	[3.0]		1			1	—
*KMT2D*	1	[3.0]			1	[1.5]	1					—
*KRAS*	6	[18.2]	5	[15.2]	11	[16.7]	11					—
*MET*	1	[3.0]			1	[1.5]	1					—
*MLH1*	1	[3.0]			1	[1.5]		1				—
*MTOR*	1	[3.0]			1	[1.5]	1					—
*NF1*	1	[3.0]			1	[1.5]		1				—
*NRAS*	2	[6.0]	3	[9.1]	5	[7.6]	5					—
*PBRM1*	6	[18.2]	4	[12.1]	10	[15.2]	5	4			1	—
*PDGFRA*	1	[3.0]			1	[1.5]	1					—
*PIK3C2A*	1	[3.0]	3	[9.1]	4	[6.1]	4					—
*PIK3C2G*	1	[3.0]	2	[6.0]	3	[4.5]	2				1	—
*PIK3CA*	2	[6.0]	5	[15.2]	7	[10.6]	6			1		—
*PKHD1*	2	[6.0]			2	[3.0]	2					—
*PLCG1*	1	[3.0]			1	[1.5]	1					—
*POLE*	1	[3.0]	2	[6.0]	3	[4.5]	3					—
*POLQ*			1	[3.0]	1	[1.5]				1		—
*PTEN*	2	[6.0]	1	[3.0]	3	[4.5]	1				2	—
*PTPN11*	1	[3.0]			1	[1.5]	1					—
*RASA*			1	[3.0]	1	[1.5]	1					—
*STK11*	1	[3.0]			1	[1.5]		1				—
*TET1*	2	[6.0]	2	[6.0]	4	[6.1]	4					—
*TGFBR2*	3	[9.1]			3	[4.5]	2				1	—
*TP53*	8	[24.2]			8	[12.1]	7		1			**0.011**

**Note:** M. missense; N. nonsense; S. splite site alteration; D. deletion; F. frameshift.

^*^Fisher test for multiple comparison was used as appropriated.

**Table 3:** Detailed distribution of copy number variations detected in 66 intrahepatic cholangiocarcinomas. Related to Fig. 2.

Gene	Poor		Good		Total		p-value*
	33	[%]	33	[%]	66	[%]	
*APC*	2	[6.1]	1	[3.0]	3	[4.5]	—
*ARID1A*	3	[9.1]	5	[15.2]	8	[12.1]	—
*CDKN2A*	12	[36.4]	12	[36.4]	24	[36.4]	—
*CCND1*	9	[27.3]	8	[24.2]	17	[25.8]	—
*EGFR*	1	[3.0]	1	[3.0]	2	[3.0]	—
*EPHA7*	4	[12.1]	6	[18.2]	10	[15.2]	—
*ERBB2*	4	[12.1]	5	[15.2]	9	[13.6]	—
*FGFR3*	9	[27.3]	5	[15.2]	14	[21.2]	—
*FHIT*	8	[24.2]	11	[33.3]	19	[28.8]	—
*KRAS*	8	[24.2]	9	[27.3]	17	[25.8]	—
*MET*	11	[33.3]	5	[15.2]	16	[24.2]	0.15
*MYC*	10	[30.0]	5	[15.2]	15	[22.7]	0.24
*PTEN*	5	[15.2]	5	[15.2]	10	[15.2]	—
*RB1*	2	[6.1]			2	[3.0]	—
*ROS*	1	[3.0]	3	[9.1]	4	[6.1]	—
*SMAD4*	4	[12.1]	4	[12.1]	8	[12.1]	—
*SRC*	8	[24.2]	10	[30.3]	18	[27.3]	—
*TP53*	7	[21.2]			7	[10.6]	**0.024**

^*^Fisher test for multiple comparison was used as appropriated.

Mutations in one or more of the 90 analysed genes were observed in 56 cases (84.8%) of our series. In detail: one mutation was observed in 26 cases (39.4%), two or more in 30 cases (45.4%) while no alteration in 10 cases (15.2%). The most frequently mutated genes across the whole cohort were *IDH1*, *KRAS* (11 mutated cases each; 16.7%), *PBRM1* (10 cases; 15.2%) and *BAP1* (9 cases; 13.6%).

CNVs for one or more of the 18 analysed genes were observed in 59 cases. In detail: one CNV was observed in 6 cases (9.1%), more than one in 46 cases (69.7%) while no CNV was observed for 7 cases (10.6%). CNV analysis showed that loss of heterozygosis (LOH) or homozygous deletion (HD) of *CDKN2A* was most frequent event (24 cases; 36.4%), followed by LOH at the *FHIT locus* (19 cases; 28.8%) and copy gain of *SRC* (18 cases; 27.3%).

To further complement mutation and CNV analysis, all cases were screened through break-apart FISH probe to detect rearrangement of *FGFR2*, *FGFR3* and *ROS1* genes. A cut-off of 20% was used to define positive specimens. Only one case showed rearrangement at the *FGFR2* gene *locus* (Supplementary Figure S2). No other rearrangement was observed for *FGFR3* and *ROS1* at the defined cut-off of 20%.

Comparing the two prognostic groups (PP and GP), different mutational rates were observed: a mean of 2.3 mutations per sample was obtained for the PP group whereas the average mutation rate was 1.5 for the GP group (Unpaired t-test *p* = 0.0007). Furthermore, one hyper-mutated sample was identified in the PP group, that is a sample characterized by a gross excess of point mutations relative to the same tumour type as analysed here and reported in current literature^15–19^. In particular, this case showed a missense mutation in *POLE*, a gene already linked to hyper-mutated genomic profiles in previous studies^20,21^. Two cases without mutations were observed in the PP group while in GP groups 8 cases showed no mutation. Differences between groups were observed in the number of CNVs as well, albeit not statistically significant. A mean of 3.3 CNVs per sample was observed in the PP group vs. a mean of 2.9 in the GP group.

As for altered genes prevalence (Fig. 2, Table 2), *TP53* was the most frequently mutated gene of the PP group (8 cases; 24.2%; *p* = 0.011) and its mutation was exclusive to this group. *ARID1A*, *IDH1*, *KRAS* and *PBRM1* displayed the second highest alteration frequency in the very same group (6 cases; 18.2%). *ARID1A* mutation was exclusive to this group as well (*p* = 0.024), and mutually exclusive with *TP53* mutation. Of note, mutations of *TGFBR2* (3 cases; 9.1%), *BRAF* and *IGF2R* (2 cases each; 9.1%) were present exclusively in the PP group, albeit at low rates. As for CNVs (Table 3), loss of one or both copies of *CDKN2A* and copy gain of *MET* (12 cases; 36.4%) were the most frequent events followed by copy gains in *MYC* (10 cases; 30.3%). LOH of *TP53* was particularly enriched in this group (7 cases; 21.2%; *p* = 0.024) and associated to mutation at the same *locus*.

The 33 patients included in GP group had exclusive mutations in *IDH2* (2cases; 3.0%), *APC*, *ARAF*, *POLQ* and *RASA* (1 case each; 1.5%), while it shared mutations in *KRAS*, *IDH1*, *BAP1* and *PIK3CA* (5 cases each; 15.2%) with the PP group. As for CNVs, the loss of *CDKN2A* was the most frequent event in the GP group (12 cases; 36.4%), a feature shared with the PP group. The second most frequent event was LOH at *FHIT locus* (11 cases; 33.3%). *MYC* copy gain (5 cases; 15.2%) was less frequently altered than in the PP group.

### Survival analysis identified markers of poor outcome and disease recurrence

Pathological features were matched to clinical data to identify poor prognostic markers. Grade, stage, sex and resection margins status were tested. Presence of positive resection margins (*p* < 0.0001) and advanced stage (*p* = 0.026) were identified as poor prognostic markers.

To investigate which of the previously identified molecular feature had the greatest impact on aggressive behaviour, we compared overall survival and progression free survival (PFS) curves in presence *vs*. absence of a specific alteration by univariate analysis.

**Figure 3:**
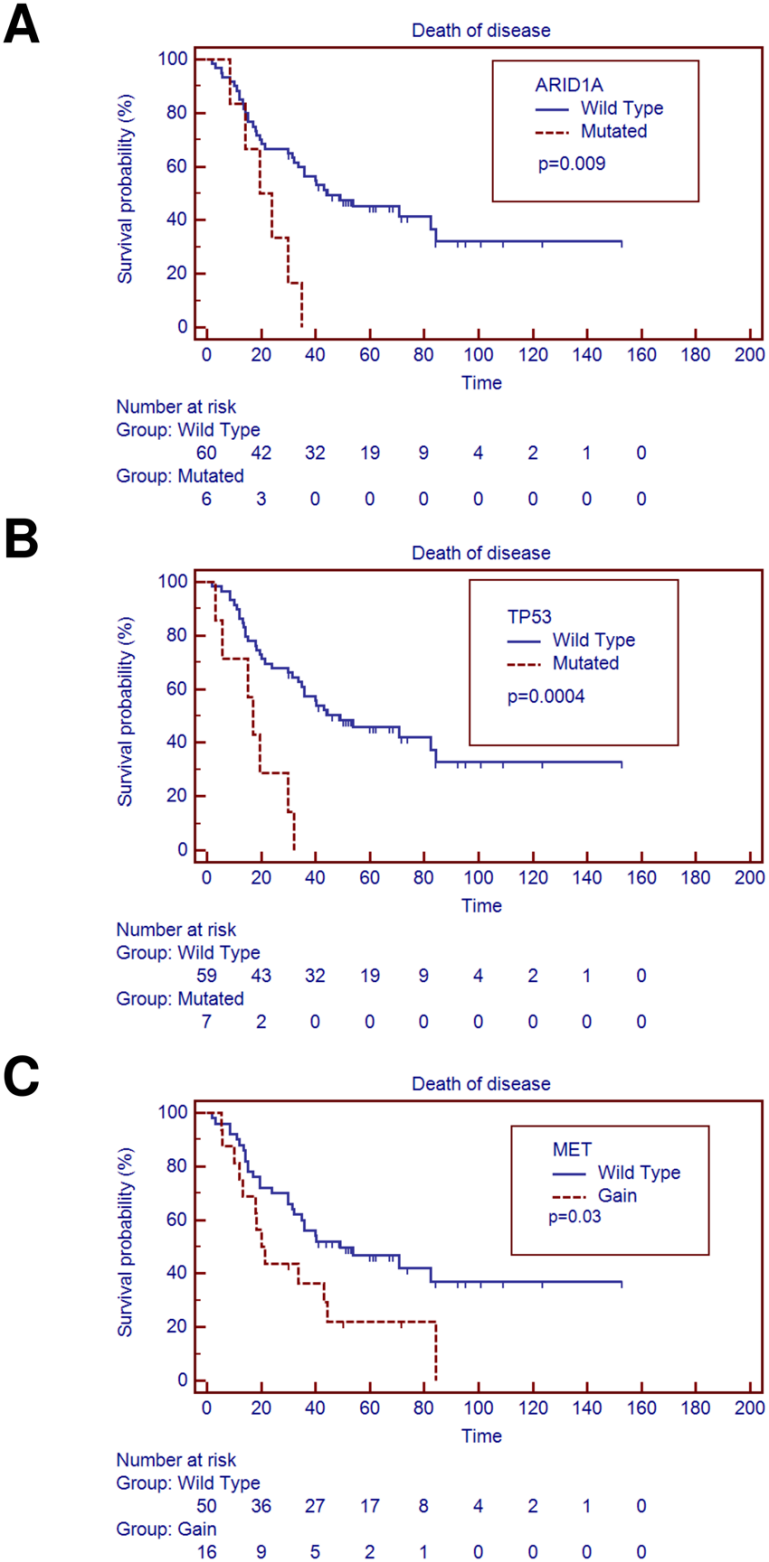
Univariate analysis identified poor prognostic markers in whole cohort. (**A**) Overall survival is significantly affected by mutations in *ARID1A* gene (*p* = 0.009); (**B**) *TP53* gene (*p* = 0.0004) mutations and (**C**) copy gain in *MET* gene (*p* = 0.03).

Considering the whole cohort, we assessed the prognostic impact of principal differently distributed molecular alterations between GP and PP groups: mutations in *TP53*; mutations in *ARID1A*; copy gains in *MET*; copy gains in *MYC*. LOH in *TP53* was not considered because already included in the *TP53* mutated cases. As illustrated in Fig. 3, alterations in *TP53* (*p* = 0.0004), *ARID1A* (*p* = 0.009) and *MET* (*p* = 0.03) genes but not gain in *MYC* gene (*p* = 0.065) were predictors of poorer prognosis at univariate analysis.

**Figure 4:**
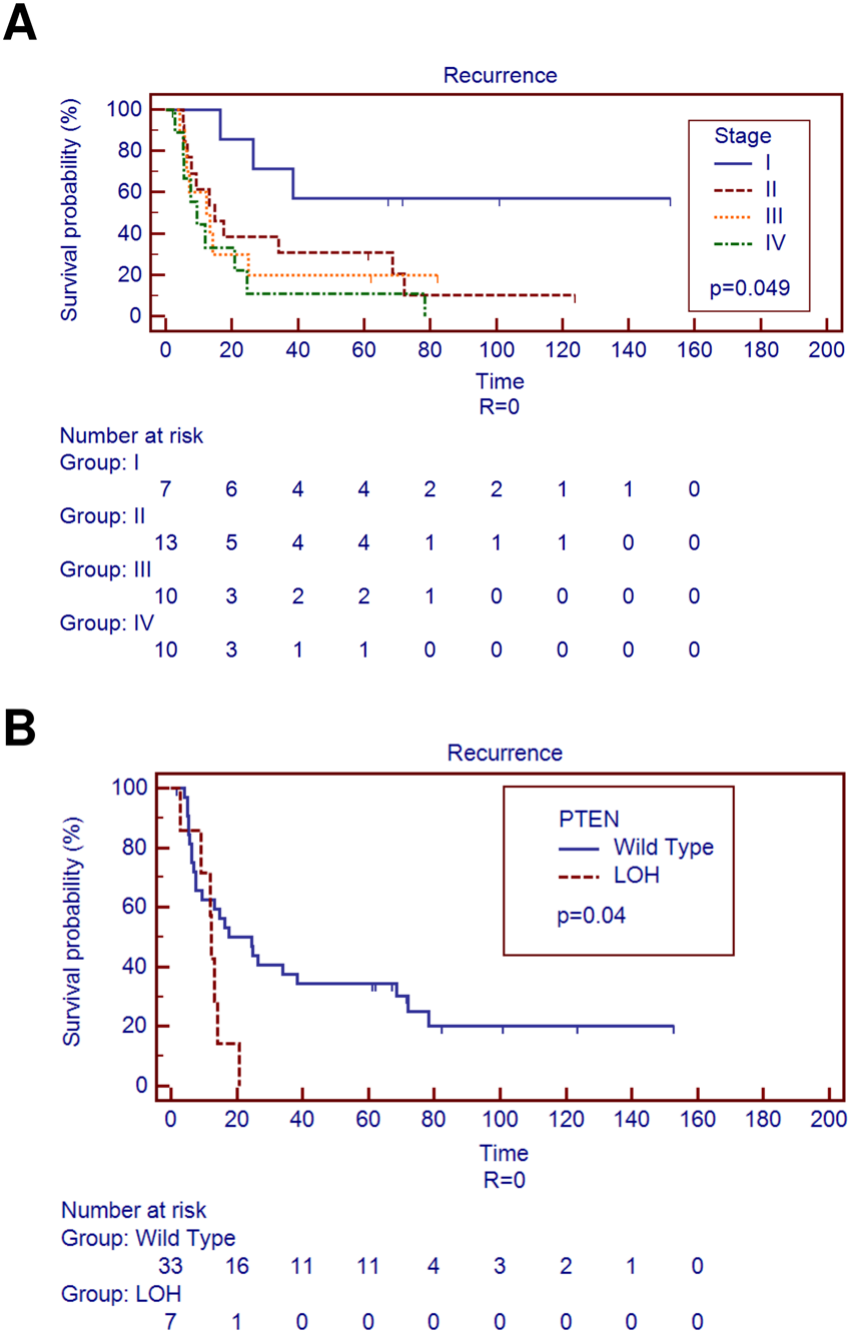
Univariate analysis identified markers of disease recurrence in negative resection margins cases. (**A**) Disease recurrence is significantly affected by advanced stages and (**B**) LOH of *PTEN* gene.

Aggressive behaviour, measured as power of relapse, was tested using disease free survival and recurrence status in the 40 patients of our cohort that displayed negative resection margins. To perform this, we compared progression free survival (PFS) of these 40 cases grouping them on the basis of their clinicopathological and molecular features. At univariate analysis, we identified advanced tumour stage (*p* = 0.049) and LOH at *PTEN* gene *locus* (*p* = 0.04) as markers of earlier disease recurrence (Fig. 4).

Cox regression for multivariate survival analysis was used to test the independent prognostic value in intrahepatic cholangiocarcinomas of the previously identified molecular markers, selecting as candidates those characterized by a *p-value* under 0.2 at univariate analysis. For clinical outcome, we tested the association of overall survival to mutation in *TP53 and ARID1A*, gains affecting *MET* and *MYC loci*, tumour stage, grade and resection margins status. Results are summarized in Table 4 and show that the resulting independent molecular predictors of poor outcome were mutation of *TP53* (*p* = 0.0031) and *ARID1A* (*p* = 0.0007), and copy gain of *MET* (*p* = 0.0003).

**Table 4:** Multivariate survival analysis of 66 intrahepatic cholangiocarcinomas; median overall survival was 40.7 months and 41 subjects died of disease.

Covariate	Odds-ratio	95% CI	p-value*
*TP53* wild type	1	—	—
*TP53* mutated	5.458	1.7729 to 16.8033	**0.0031**
*ARID1A* wild type	1	—	—
*ARID1A* mutated	6.9059	2.2710 to 21.0008	0.0007
*MET* wild type	1	—	—
*MET* gain	4.6312	2.0210 to 10.6126	**0.0003**
*MYC* wild type	1	—	—
*MYC* gain	1.6521	0.7391 to 3.6929	0.2212
Stage I	1	—	—
Stage II	1.8791	0.4878 to 7.2383	0.3592
Stage III	7.4306	2.0001 to 26.6060	**0.0027**
Stage IV	2.7035	0.7327 to 9.9752	0.1354
Grade 1	1	—	—
Grade 2	0.7426	0.1978 to 2.7885	0.6593
Grade 3	2.09	0.5534 to 7.8932	0.2769
Negative resection margins	1	—	—
Positive resection margins	4.4314	1.4418 to 13.5611	**0.0091**

^*^Cox proportional-hazards regression analysis. Selection of the best model was performed using the “forward” algorithm.

### Protein expression evaluation of principal markers

Immunohistochemical analysis was performed on all samples included in the study to evaluate protein expression level of candidate gene markers previous identified. We observed positive stain for p53 in all samples affected by missense mutation, while the case affected by a nonsense mutation showed no protein expression (Supplementary Figure S3A).

Similarly, arid1a immunolabelling was negative in presence of nonsense and frameshift mutations but not when samples were unaffected or affected by missense mutations (Supplementary Figure S3B).

As for pten protein, we observed a generally low or negative immunolabelling irrespective of the presence of LOH, mutation, or no alteration at all.

Finally, we observed positive immunolabelling for c-met protein in those cases which had the MET *locus* affected by copy gain (Supplementary Figure S3C).

## Discussion

The incidence and mortality rates of ICC have been rising worldwide in the past decade^2^ and only 10–20% of patients present with an early-stage disease amenable to curative surgery^4,5^. Molecular analyses have suggested that the observed heterogeneity in prognosis and response to treatments could be attributed to the underlying molecular mechanisms driving crucial differences in cancer aggressiveness and treatment outcomes^11^.

To date, many studies have been performed to uncover molecular features characterizing the different subtypes of cholangiocarcinoma and to infer mechanisms underlying its aggressive behaviour. Recently, two multi-omics studies were performed in unbiased large cohorts of cholangiocarcinomas identifying poor prognostic hallmarks^15,19^. In particular, Nakamura *et al*. identified shorter survival in a group characterized by a higher mRNA expression of immune checkpoint genes^15^ while, Jusakul *et al*. identified the presence of liver fluke infection as a marker for poor prognosis^19^. In both studies a heterogeneous cholangiocarcinoma cohort (including gallbladder, extrahepatic and intrahepatic tumours) was used and survival analysis was performed on groups characterized by concomitant multiple alterations. Identification of these simultaneous alterations in diagnostic routine remains expensive and difficult to apply on archive material. Focusing on the intrahepatic subtype, to date several molecular studies were performed. In these, aside from mutations in *TP53*^14,15,22,23^, other different molecular alterations were identified as poor prognosis markers as *IDH1*^12^, *RNF43*^24^, *KRAS* and *ARID2*^15^. In all these studies, the experimental rationale was to start from a histopathologically defined cohort to identify new molecular and prognostic markers.

In this study, we followed a different experimental approach, using HCTS to analyse genetic alterations in two cohorts of ICC previously grouped according to their different prognostic performance. The idea of molecularly characterizing different prognostic groups in order to investigate the potential existence of different driver alterations is innovative and promising, yet conceptually similar to treatment response trials or previous research works where clinicopathological variables were compared between 2 subgroups of patients distinct according to treatment response^25^ or prognosis^26^.

We selected and grouped 66 patients in two sub-cohorts according to the clinical performance showed within 36 months: PP group (dead of disease at <36 months) and GP group (alive at >36 months). We confirmed a statistically significant difference between the two groups both for the prognostic performance in all cases (*p* < 0.0001) and for disease recurrence in negative resection margin cases (*p* < 0.0001).

Sequencing analysis evidenced different mutational rates between groups (*p* = 0.0033), with poor prognostic performers bearing a higher number of mutations, in keeping with a recent study in which a hyper-mutated profile was associated to poorer prognosis^15^. Mutational analysis identified *IDH1*, *BAP1*, *PBRM1* and *KRAS* as the most frequently altered genes, as in previous whole-exome and whole-genome sequencing studies^12,13,15^. Of note, coding sequence mutations of *TP53* were found only in poor prognostic performers in our study, with a significant discrepancy (*p* = 0.011) between groups. Similarly, we found mutations of *ARID1A* only in this prognostic group (*p* = 0.024). The association between mutations in *TP53* and poor prognosis is not novel for intrahepatic cholangiocarcinomas^15,22,23,27^. In particular, Jayle *et al*. showed similar results in a large unbiased group of 224 ICCs where *TP53* resulted mutated in 24% of cases^23^. Differently, to date only low expression of *ARID1A* protein and mRNA were associated to poor prognosis in 57 intrahepatic cholangiocarcinomas analysed by Yang and colleagues. Although this corroborates our finding in suggesting a prognostic role of *ARID1A* in ICC, unfortunately no mutational analysis was performed in that study^28^. Furthermore, a recent meta-analysis of Luchini *et al*. showed as mutation or low expression of *ARID1A* is a predictor of shorter disease specific survival and time to disease recurrence in cancer patients, but a direct correlation to ICC was not evident^29^.

Copy number variation analysis identified LOH in *CDKN2A* as the most frequent event, in keeping with previous studies^15,30^, with no difference in alteration rates between the two groups. Conversely, a higher proportion of cases affected by copy gain in *MET* and in *MYC* genes was observed in the PP group. The association between *c-MET* and poor prognosis is not novel, as a previous study showed by immunohistochemistry that overexpression of this proto-oncogene is correlated to poorer outcome in patients affected by cholangiocarcinoma^31^. However, we herein show for the first time that patients affected by *MET* and *MYC* gene copy gain associate to a worse prognosis, suggesting that the enhanced expression of these *loci* may be due to a genetic lesion at least in a fraction of cases.

When we considered only cases with negative resection margins, only alterations affecting *PTEN* showed a prognostic impact on disease recurrence. Recently, heterozygous and homozygous deletion of *PTEN locus* in presence of *KRAS* activation was demonstrated to induce intrahepatic cholangiocarcinoma in cholangiocytes of a new mouse model^32^ showing an important role of *PTEN* in the development of this tumour type.

The main strength of the present work was the selection and focus on a well-defined cohort of resected ICC cases, grouped by prognostic performance. This however leads directly to the main limitation of the study, that suffers from a relatively small sample due to the difficulties in enrolling large numbers of resected patients with a minimum follow-up of 3 years, availability of material for research and no preoperative therapy. Despite that, our study shows a clear overlap with previous studies when dealing with already known associations (e.g. *TP53* mutation), while providing several interesting evidences about independent poor prognostic markers for intrahepatic cholangiocarcinomas. The preliminary stratification of patients according to prognosis also allowed the identification of some molecular aberrations whose absence in the GP group could explain an unusual degree or duration of the clinical benefit in selected cases of intrahepatic cholangiocarcinoma, as opposed to an otherwise relatively ineffective treatment in the rest of patients. The present work therefore confirms previous knowledge on the molecular landscape of ICC and suggests potential hallmarks of aggressiveness in this tumours that demand further validation but may be easily translated to the clinic for anticipating prognosis and response to therapy.

## Materials and Methods

### Patients and samples enrolled in the study

Tissue specimens and data from surgically-resected primary intrahepatic cholangiocarcinoma patients were retrieved from the ARC-Net Biobank at Verona University Hospital. Three criteria were considered to enrol patients in this study: i) minimum follow-up of 3 years; ii) availability of material for research; iii) no preoperative therapy received. According to the stated criteria, a retrospective series (1990–2013) of 66 surgically-resected primary intrahepatic cholangiocarcinomas (ICC) was retrieved from the FFPE archives of the biobank under the local ethics committee approval (“Comitato etico per la sperimentazione clinica delle province di Verona e Rovigo” n. prog. 1959). All cases were reclassified by two pathologists (MF and AS) according to WHO 2010^33^ and staged according to AJCC/UICC 7th edition^34^. In all cases, sufficient material for molecular analysis and construction of 1-mm cores tissue microarrays (TMAs) was available. Three tissue cores per case were included in the TMAs. Eighteen non-neoplastic samples (8 normal biliary duct and 10 chronic cholecystitis) were included in the TMAs as controls.

### DNA extraction and qualification

DNA from tumour and matched non-neoplastic liver was extracted from formalin-fixed paraffin embedded (FFPE) tissue specimens. In particular, tumour DNA was prepared after enriching neoplastic cellularity to at least 70% by manual microdissection of 10 consecutive 4-μm sections: cases were revised by a pathologist that chose tissue areas according to two main features: i) absence of necrosis and ii) tissue histology had to be representative of the whole tumour. DNA was purified using the QIAamp DNA FFPE Tissue Kit (Qiagen), and qualified as reported elsewhere^35,36^.

### Mutational analysis by next-generation targeted sequencing

Matched tumour/normal DNA from all cases was subjected to targeted next-generation sequencing (NGS). Two multigene panels were used to investigate mutational status of 90 genes: the 50-gene Ion AmpliSeq Cancer Hotspot panel v2 (Thermo Fisher) and one AmpliSeq custom panel (ICC custom panel 1) targeting 40 genes not included in the commercial panel. The first explores selected regions of 50 cancer-genes: *ABL1*, *AKT1*, *ALK*, *APC*, *ATM*, *BRAF*, *CDH1*, *CDKN2A*, *CSF1R*, *CTNNB1*, *EGFR*, *ERBB2*, *ERBB4*, *EZH2*, *FBXW7*, *FGFR1*, *FGFR2*, *FGFR3*, *FLT3*, *GNA11*, *GNAS*, *GNAQ*, *HNF1A*, *HRAS*, *IDH1*, *IDH2*, *JAK2*, *JAK3*, *KDR/VEGFR2*, *KIT*, *KRAS*, *MET*, *MLH1*, *MPL*, *NOTCH1*, *NPM1*, *NRAS*, *PDGFRA*, *PIK3CA*, *PTEN*, *PTPN11*, *RB1*, *RET*, *SMAD4*, *SMARCB1*, *SMO*, *SRC*, *STK11*, *TP53*, *VHL*. Details on target regions of the commercial panel are at http://www.thermofisher.com. The custom panels targets 40 genes selected on the basis of published WGA, exome and targeted sequencing studies: *ACVR2A*, *ARAF*, *ARID1A*, *ARID1B*, *ARID2*, *ATR*, *BAP1*, *CHD4*, *DNMT3A*, *ELF3*, *EP300*, *EPHA2*, *EPHA6*, *EPHA7*, *GSK3A*, *HERC2*, *KMT2A*, *KMT2C*, *KMT2D*, *MDC1*, *MSH6*, *MTOR*, *NF1*, *PBRM1*, *PIK3C2A*, *PIK3C2G*, *PKHD1*, *PLCG1*, *POLE*, *POLQ*, *PRKDC*, *RASA*, *RELN*, *RNF43*, *ROBO2*, *ROS*, *SF3B1*, *TET1*, *TET2*, *TGFBR2*^12–15,24^. Details on target regions of the commercial panel are at http://www.thermofisher.com while detail of region covered by ICC custom panel 1 are reported in Supplementary Table S4. Twenty ng of DNA were used for each multiplex PCR amplification. The quality of the obtained libraries was evaluated by Agilent 2100 Bioanalyzer on-chip electrophoresis (Agilent Technologies). Emulsion PCR for clonal amplification of the libraries was performed with the Ion OneTouch™ OT2 System (Thermo Fisher). Sequencing was run on the Ion Proton (PI, Thermo Fisher) loaded with Ion PI Chip v2. Data analysis, including alignment to the hg19 human reference genome and variant calling, was done on the Torrent Suite Software v.5.0 (Thermo Fisher) utilizing the standard setup for somatic variants detection with minor modifications to further improve sensitivity (i.e. minimum variant frequency for indels was set to 0.05 instead of 0.15; complex variant detection was enabled). Called variants were annotated using a custom pipeline based on vcflib (https://github.com/ekg/vcflib), SnpSift^37^, the Variant Effect Predictor (VEP) software^38^ and NCBI RefSeq database. Confident somatic mutations were obtained from annotated variants by: i) filtering-out germline mutations identified in matched normal sample sequenced; ii) filtering-in mutations with at least 20 variant-containing reads and with variant frequency >10%; iii) ruling out sequencing artefacts by visual verification of normal and tumour samples alignments using the Integrative Genomics Viewer (IGV) v2.3^39^. Alignments were visually verified with IGV also to confirm the presence of identified mutations.

### Copy number variations of cholangiocarcinomas by next-generation sequencing

A second custom panel was specifically developed to investigate CNV status of 18 genes: *APC*, *ARID1A*, *CDKN2A*, *CCND1*, *EGFR*, *EPHA7*, *ERBB2*, *FGFR3*, *FHIT*, *KRAS*, *MYC*, *MET*, *PTEN*, *RB1*, *ROS*, *SMAD4*, *SRC* and *TP53* (ICC custom panel 2, Supplementary Table S4). CNV analysis was performed on IonReporter Software v.5.0 (Thermo Fisher) utilizing the single-sample CNV workflow. According to this workflow, a baseline was created using the alignment files of 10 DNA samples from FFPE tissue of male healthy donors. Alignment files of tumour samples were then compared to the CNV baseline to determine CNV status. CNV calls were deemed confident according to the following criteria: i) a CNV confidence number major than 20; ii) a tiles number major than 10. For genes showing CNV calls with sub-optimal values, an orthogonal cross-validation using FISH or qPCR was performed. In this case, only CNV calls with concordant results of NGS and validation were reported. A statistical report of cross-validation rates is illustrated in Supplementary Table S5.

### CNV validation by Quantitative PCR

Q-PCR analysis of copy numbers was applied to all samples for selected loci. All target and reference assays were purchased from Applied Biosystems. *RNaseP* was used as endogenous control for normalization of analysed loci. The following assays were used: *CCND1* (Hs03772544), *FHIT* (Hs03491211), *MET* (Hs04951661), *SRC* (Hs07169853) and *RNaseP* (part number 4403326). The experimental procedure recommended by the manufacturer (Applied Biosystems) was followed. Twenty ng of genomic DNA were used in the q-PCR reaction and a negative control was analysed in parallel. All q-PCR reactions were run in quadruplicate in a 7900HT qRT-PCR machine (Applied Biosystems) using standard cycling conditions of 10 min at 95 °C, followed by 40 cycles of [95 °C for 15 sec and at 60 °C for 1 min]. Pooled normal FFPE DNA was used as calibrator.

### Fluorescent *in situ* hybridization (FISH)

A FISH analysis was developed according manufacturer instruction for following genes to validate Copy Number Variation obtained by NGS analysis: *CDKN2A*, *EGFR*, *ERBB2*, *FGFR3*, *MYC*, *MET*, *PTEN*, *ROS* and *TP53* (all probes Vysis/Abbott Molecular). Analysis was performed for all samples; data interpretation was performed as reported elsewhere^40–43^ and cross-validation rates are illustrated in Supplementary Table S5. FISH analysis was also used to identify rearrangement for followed genes: *FGFR2* (ZytoVision Molecular Diagnostics), *FGFR3* (CGI) and *ROS1* (Vysis/Abbott Molecular). FISH analysis was firstly performed on TMA for all cases. Cases showing a positive signal in at least 15% of nuclei were re-analysed by FISH on whole sections. A presence of rearrangement in at least 20% of cells was considered positive. Representative cases of *PTEN* and *TP53* monosomy and *CDKN2A* homozygous deletion are illustrated in Supplementary Figure S1, while the only case with confirmed *FGFR2* rearrangement is shown in Supplementary Figure S2.

### Immunohistochemistry

The immunohistochemical staining was performed with a Leica Microsystems Bond-Max Autostainer System according to manufacturer protocols. Arid1a (clone EPR13501–73, Abcam, dilution 1:1000), met (clone 4F8.2, Sigma Aldrich, dilution 1:250), pten (clone 138G6, Cell Signaling, dilution 1:100) and p53 (clone DO-1, Immunotech, dilution 1:50) were applied to consecutive 4-μm FFPE TMA sections. Appropriate positive and negative controls were run concurrently. Representative cases altered in p53, arid1a and met are shown in Supplementary Figure S3.

### Statistical analysis

One-way ANOVA, Kruskal-Wallis test, Fisher’s test with Monte Carlo simulation, and Fisher’s exact test corrected for multiple comparisons were used as appropriate. For comparison of Kaplan-Meier survival curves, Mantel-Cox log-rank test was used; for multivariate survival analysis, stepwise Cox proportional hazards regression was used; selection of the best model was performed using the “backward elimination” algorithm. For all the analyses, a *p-value* below 0.05 was considered significant. All analyses were performed using Medcalc for Windows version 15.6 (MedCalc Software, Ostend, Belgium) and R v. 3.2.1; multivariate Cox regression was done with R using survival library v.2.38-2.

## Electronic supplementary material

Supplementary Figures

Supplementary Table S1

Supplementary Table S2

Supplementary Table S3

Supplementary Table S4

Supplementary Table S5
