# Supplementary material for: Genetic alterations analysis in prognostic stratified groups identified TP53 and ARID1A as poor clinical performance markers in intrahepatic cholangiocarcinoma
Source: Sci Rep. 2018 May 8;8:7119. doi: 10.1038/s41598-018-25669-1 (PMC5940669; doi:10.1038/s41598-018-25669-1)
Supplement: Supplementary file 1 — Supplementary Figures [file 41598_2018_25669_MOESM1_ESM.pdf]

– Supplementary Information –

Genetic alterations analysis in prognostic stratified groups identified *TP53* and *ARID1A* as poor clinical performance markers in intrahepatic cholangiocarcinoma

Michele Simbolo[1,2], Caterina Vicentini[1,2], Andrea Ruzzenente[3], Matteo Brunelli[2], Simone Conci[3], Matteo Fassan[1°], Andrea Mafficini[1], Borislav Rusev[1,2], Vincenzo Corbo[1,2], Paola Capelli[2], Emilio Bria[4], Serena Pedron[2], Giona Turri[2], Rita T. Lawlor[1,2], Giampaolo Tortora[4], Claudio Bassi[5], Alfredo Guglielmi[3], Aldo Scarpa[1,2]

1 ARC-Net Research Centre, University and Hospital Trust of Verona, Verona, Italy

2 Department of Diagnostics and Public Health, Section of Anatomical Pathology, University and Hospital Trust of Verona, Verona, Italy

3 Department of Surgery, General and Hepatobiliary Surgery, University Hospital G.B. Rossi, University of Verona

4 Department of Medicine, U.O.C Oncology, University and Hospital Trust of Verona, Verona, Italy

5 Department of Surgery and Oncology, Pancreas Institute, University of Verona, Verona, Italy

° Present address: Department of Medicine (DIMED), Surgical Pathology and Cytopathology Unit, University of Padua, Padua, Italy

Correspondence to: Prof Emilio Bria, MD, Department of Medicine, U.O.C Oncology, University and Hospital Trust of Verona, Verona, Italy Piazzale L.A. Scuro, 10, Tel.: +390458128124, fax: +390458027410, email: emilio.bria@univr.it

Supplementary Figures: 3

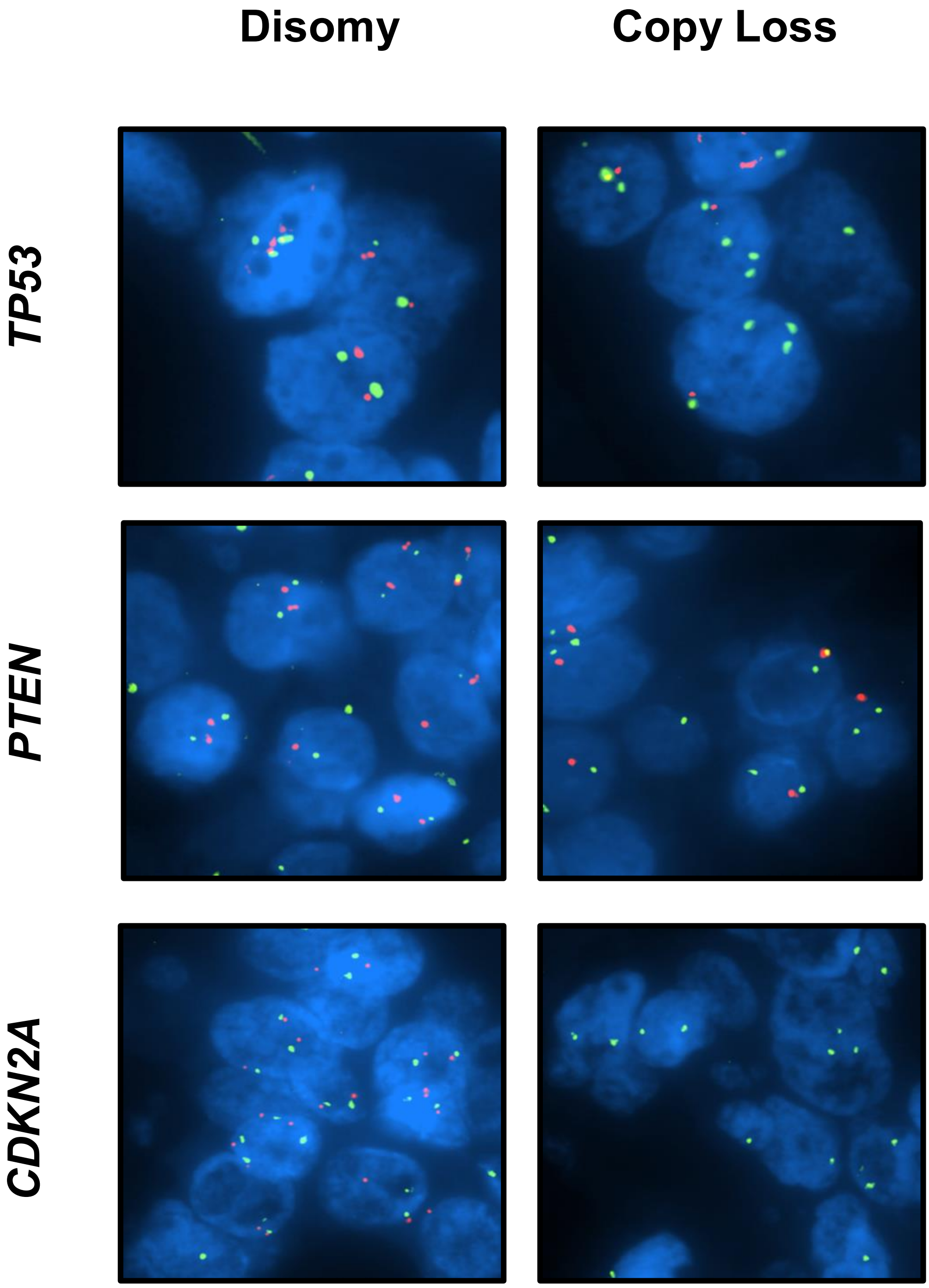

Supplementary Figure S1: FISH validation of copy number variations. Representative cases affected by loss of one or both gene copies of **A)** *TP53*, **B)** *PTEN* and **C)** *CDKN2A* are illustrated and compared to cases showing disomy for the same target.

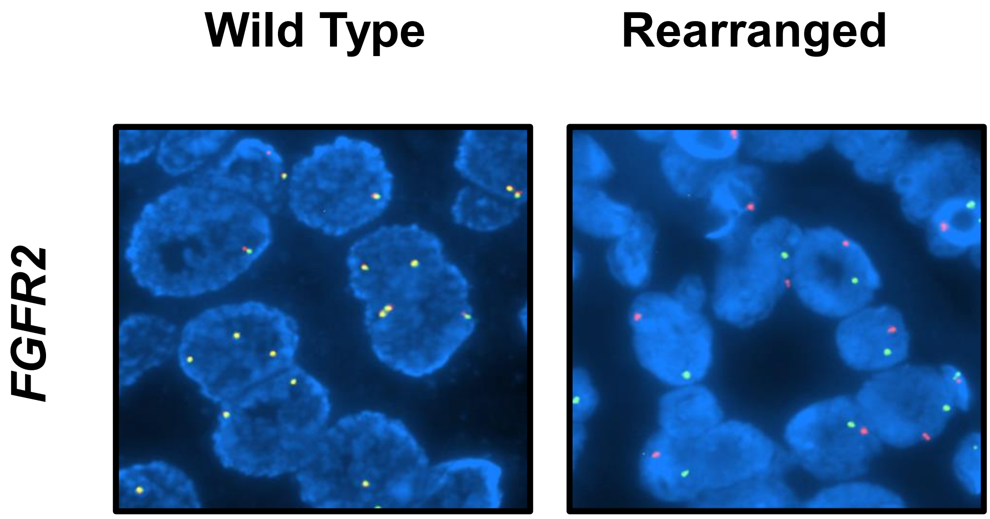

Supplementary Figure S2: Representative image of *FGFR2* rearrangement. Representative images of a wild-type (left) intrahepatic cholangiocarcinoma with Intact FISH probes (yellow colour), and of the rearranged (right) case with split FISH probes (red/green colours).

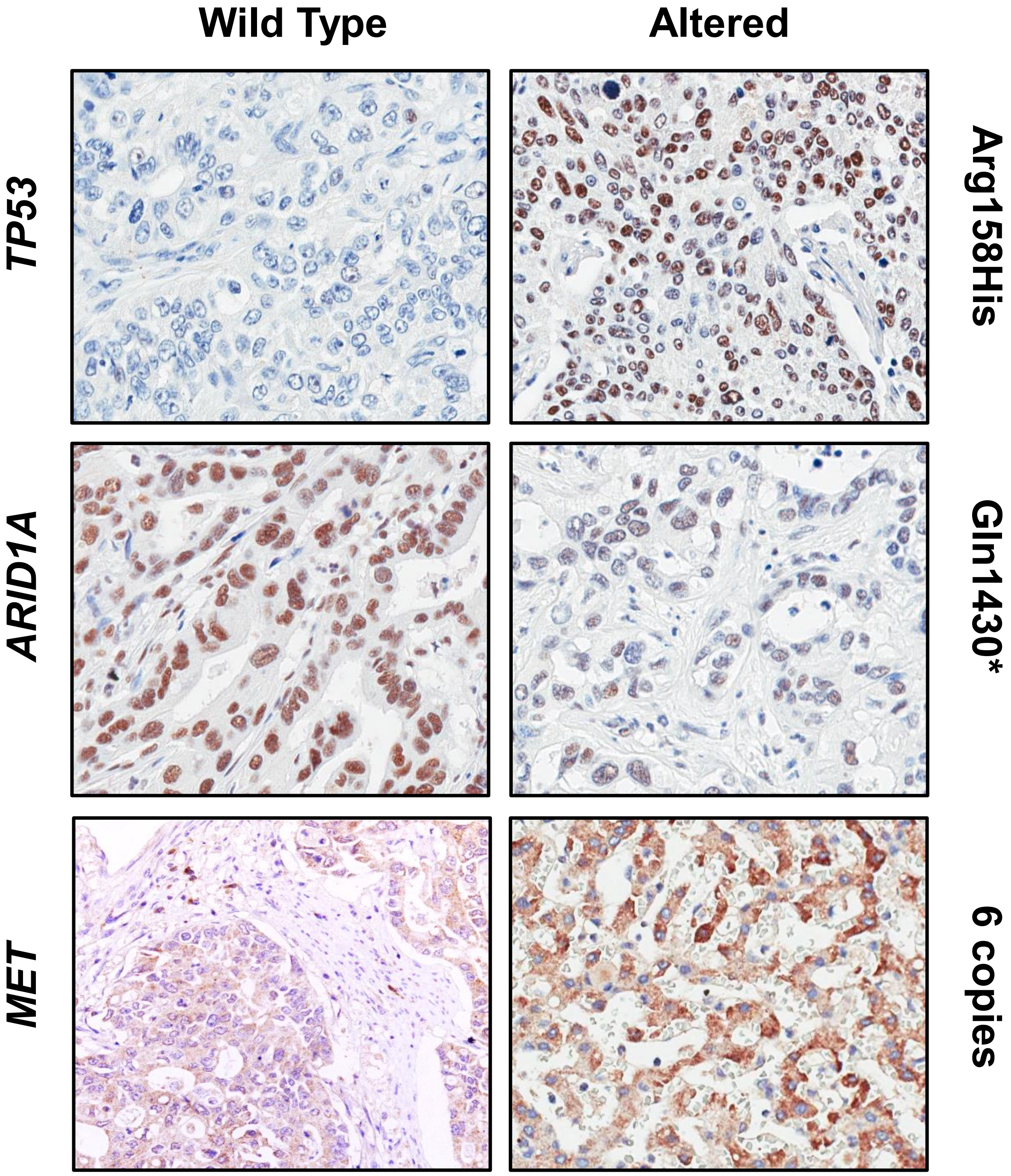

Supplementary Figure S3: Immunohistochemical analysis. Representative cases with no alteration in genes evaluated on the left while three cases characterized by alterations (mutation of *TP53* and *ARID1A* and copy gain of *MET*) on the right. The specific alteration affecting each case is reported on the right.
